# Co-Designing a Conversational Agent With Older Adults With Chronic Obstructive Pulmonary Disease Who Age in Place: Qualitative Study

**DOI:** 10.2196/63222

**Published:** 2024-10-08

**Authors:** Emilie Kauffeldt Wegener, Jenny M Bergschöld, Tina Kramer, Camilla Wong Schmidt, Karen Borgnakke

**Affiliations:** 1 Department of Public Health University of Copenhagen København Denmark; 2 Department of Health SINTEF Digital Oslo Norway; 3 Region Zealand Sorø Denmark

**Keywords:** eHealth, aging in place, digital health technology, health literacy, everyday life, co-design, co-designing, conversational agent, older adults, elderly, COPD, thematic analysis, design, development, interview data, cocreation, chronic obstructive pulmonary disease, mobile phone

## Abstract

**Background:**

As a reaction to the global demographic increase in older adults (aged 60+ years), policy makers call for initiatives to enable healthy aging. This includes a focus on person-centered care and access to long-term care for older adults, such as developing different services and digital health technologies. This can enable patients to engage in their health and reduce the burden on the health care systems and health care professionals. The European Union project Smart Inclusive Living Environments (SMILE) focuses on well-being and aging in place using new digital health technologies. The novelty of the SMILE project is the use of a cocreational approach focused on the needs and preferences of older adults with chronic obstructive pulmonary disease (COPD) in technology development, to enhance access, adaptation, and usability and to reduce stigma.

**Objective:**

The study aimed to describe the perspective, needs, and preferences of older adults living with COPD in the context of the design and development of a conversational agent.

**Methods:**

This study carried out a data-driven thematic analysis of interview data from 11 cocreation workshops with 33 older adults living with COPD.

**Results:**

The three particular features that the workshop participants wanted to implement in a new technology were (1) a “my health” function, to use technology to manage and learn more about their condition; (2) a “daily activities” function, including an overview and information about social and physical activities in their local area; and (3) a “sleep” function, to manage circadian rhythm and enhance sleep quality, for example, through online video guides. In total, 2 overarching themes were identified for the 3 functions: measurements, which were actively discussed and received mixed interest among the participants, and health literacy, due to an overall interest in learning more about their condition in relation to everyday life.

**Conclusions:**

The future design of digital health technology must embrace the complexities of the everyday life of an older adult living with COPD and cater to their needs and preferences. Measurements should be optional and personalized, and digital solutions should be used as a supplement to health care professionals, not as substitute.

## Introduction

### Background

Global policy makers have demanded maintained action and initiatives to enable healthy aging in all parts of the world. The United Nations and the World Health Organization (WHO) describe healthy aging as “developing and maintaining the functional ability that enables well-being in older age” [1]*.* The proportion of older adults will double from 1 billion to 2.1 billion between 2019 and 2050 globally [1]. As a reaction to this demographic change, areas of action suggested in the *UN Decade of Healthy Ageing: Plan of Action 2021-2030* include person-centered care and access to long-term care for older adults, such as developing different services including assistive technologies [1].

### Cocreating Technology With Older Adults

The European Union Horizon 2020 project Smart Inclusive Living Environments (SMILE), a collaborative project among Denmark, Norway, Canada, the Netherlands, and Greece, draws on practical situations that older adults face in daily life to create smart living environments (SLEs) with novel eHealth solutions. The project intends to assist older adults with chronic obstructive pulmonary disease (COPD) to maintain their independence in the comfort of their own homes. The project emphasizes the involvement of older adults by identifying their needs and preferences and by involving them in co-designing and testing a new technological solution [2]. The technological solution that is cocreated with end users in the SMILE project is a conversational agent (CA), designed to facilitate daily communication online through a weblink that can be accessed on a smartphone, tablet, or computer.

In SMILE, qualitative cocreation workshops are used to enhance the practical involvement of older adults in the development of technology. This highlights one of the novelties in the SMILE project, as earlier cocreation or participatory design processes have not succeeded in facilitating active participation in all phases of a design process, from the initial needs identification to the final evaluation phase [2]. The aim of the cocreation workshops is to provide a secure environment where participants can share their daily experiences and thoughts about their health conditions, preferences, needs, and daily activities. These workshops also serve as exploratory sessions for both the participants and the researchers in facilitating the generation of ideas for future technology design and development [3].

### The Living Labs

The SMILE project uses the concept of “living labs” to study the use and impact of new digital technology on older adults’ daily lives. A “living lab” is a user-centered, open ecosystem that enables the integration of research and innovation processes into real-life communities and settings [4]. Using living labs has the advantage of being independent of existing economic models and policies, making it possible to challenge existing societal structures by providing suitable arenas for testing new ideas [5].

The Danish SMILE living lab “PreCare,” was launched in 2018 and operates in a collaboration between the Odsherred municipality and Region Zealand. PreCare offers digital and outward-bound homecare treatments to older adults living with COPD and has approximately 150 older adults enrolled. PreCare works in dynamics between innovation, development, implementation, operation, and analysis based on an action research–based “plan-do-study-act cycle” [6] and is therefore in constant development. The Norwegian SMILE living lab is located in Gudbrandsdalen, a rural valley consisting of 12 municipalities. In Norway, the number of people over the age of 80 years will almost double in rural areas by 2040 [7,8], posing significant challenges in the context of demographic aging. In rural Norway, the long distances between people, workplaces, public services, care services, and institutions are challenging for older adults who age in place, especially those who have chronic conditions [9]. In SMILE, these challenges are used to collaborate closely with older adults with COPD, engaging them as co-designers and using qualitative methods to explore their lived experiences and needs [10].

### Interprofessional Collaboration

With the SMILE project’s intent, interprofessional collaboration is promoted, and existing structures and roles among patients, health care professionals, and informal caregivers are challenged [11]. Older adults, their social network, and health care professionals must be capable and willing to use these new technologies for the “smart inclusive living environment” to fully work.

Traditionally, health care professionals provide care to patients, with minimal involvement of the patients. This has gradually changed to an increased collaboration among health care professionals, patients, and informal caregivers. In the WHO’s global strategy on integrated and people-centered health services (2016-2026), people, patients, and communities are placed at the center of health services. The strategy emphasizes that the care should be organized around the health needs and expectations of people and communities rather than a specific disease. People have individual preferences, needs, and abilities, and therefore should not be understood as patients defined by their disease [12]. Therefore, innovative initiatives toward more patient-oriented activities in health care are needed, such as the co-design of new digital solutions as carried out in the SMILE project, as well as the development of new digital solutions to support both patients, citizens, and formal and informal caregivers.

## Methods

### Overview

The empirical data presented and analyzed in this study were collected through 11 cocreational workshop sessions in Denmark and Norway with people living with COPD. The analysis was thematic and privileged the participants’ descriptions and perspectives that were related to their reasons and relevance criteria for the design and use of the CA.

### Aim

The aim is to describe the perspective, needs, and preferences of older adults living with COPD in the context of the design and development of a CA.

### Participants, Recruitment, and Eligibility

A total of 33 older adults living with COPD participated in the SMILE cocreation workshops. In Denmark, 21 participated, and in Norway, 12 participated. Participants were recruited through health care partners as a part of their enrolment in the SMILE living labs. Access to the participants was established through the registered nurses at each living lab, and participants were then contacted through email or phone by the researchers. Participants were eligible to participate if they were aged 65 years or older, lived with COPD, and were able to fill in the Readiness and Enablement Index for Health Technology (READHY) instrument and take an active part in the cocreational design workshops and evaluation. Oral and written consent was obtained from all participants before the workshops (Multimedia appendix 1).

The saturation or information power of the sample size in this study was addressed based on the 5 items impacting information power in qualitative research presented by Malterud et al [13]. These included the following: (1) the study aim is specific and therefore can hold fewer participants, with its focus on identifying needs and preferences and with the purpose of cocreating a technology, through an iterative process; (2) the sample specificity is relevant to the study aim and is limited to older adults with COPD, but has variations regarding technology readiness; (3) theoretical frameworks included READHY and concepts presented by Yock et al [14] to cocreate technology with end users; (4) the dialogue with the participants was clear and followed a semistructured interview guide, which often requires fewer participants; and (5) the strategy for the analysis is an in-depth analysis of themes and narratives, for example, not a cross-case analysis where more participants often would be needed [13]. Based on these, saturation for this study was achieved.

### Ethical Considerations

Informed consent was obtained from all humans participating in this study. Data obtained from participants was handled according to General Data Protection Regulation (GDPR). Our research has followed the declaration of Helsinki’s ethical principles for medical research involving human participants. In Denmark, health science questionnaire surveys and interview studies that do not involve human biological material (section 14(2) of the Danish Act on Committees) do not require reporting or approval from the Danish National Centre for Ethics [15]. In Norway, the field studies were approved by the data protection officer at Innlandet Hospital Trust (journal 14832226).

### Data Collection

#### Cocreation Workshops

The empirical data were gathered through 11 cocreation workshops: 5 in Denmark and 6 in Norway. Each workshop had a duration of 2 hours, and between 2 and 5 participants were present at each workshop. The CA was developed through 3 design iterations and the workshops were conducted before each iteration, meaning that there were 3 phases of the workshops. Each workshop phase had different purposes, which corresponded to the development of the CA.

The purpose of the first phase of the workshops was to elaborate on early ideas, to present and discuss 9 possible functions, and to narrow these down to 3 or 4 prioritized functions based on input from the participants. In total, 4 prioritized functions were identified in the first phase of the workshops, which were (1) physical activity, (2) clinical condition, (3) social activity, and (4) sleep. These 4 functions were then implemented in a CA mock-up. After each workshop, a summary report was written and provided to the technology developers, to inform the development of the CA based on input from the participants.

The purpose of the second phase of the workshops was to present the CA mock-up to the participants and gather input that could be used to develop the CA prototype version 1. The mock-up presented to the participants was built in Botpress [16] and consisted of a basic chatbot with predetermined dialogues that concentrated on the 4 functions identified as relevant by the participants during the first phase of the workshops. The functions and mock-up were discussed with the workshop participants. This phase resulted in changing the features, that is, social activity and physical activity were merged into one feature called “daily activity.” There were changes to the interface and the conversation strings, and an onboarding feature that teaches users how to interact with the CA was included.

The third phase of the workshops was used to elaborate on the participants’ perceptions of the content and functions of the CA prototype version 1. The prototype contained an onboarding feature and three main functions: (1) my health, (2) daily activity, and (3) sleep, which were developed based on input from the participants from phase 2. Findings from the third phase were used to develop the CA prototype version 2.

All workshops were audiotaped. Before data from the workshops were analyzed, sound recordings from the cocreation workshops were transcribed verbatim in the native languages at each living lab site [17]. In analyzing the transcriptions, relevant parts presented in this study were translated into English. EKW carried out the transcription and translation of the Danish material, and JMB carried out the transcription and translation of the Norwegian material. All translations privilege clarity of meaning over the verbatim transcripts.

#### Thematic Analysis

This study conducted a thematic analysis of the data from 11 cocreation workshops. The thematic analysis was inspired by Braun and Clarke [18]; concepts of data-driven analysis; and a realist approach to give voice to the data and the realities, meanings, and experiences presented in the data. As a part of the workshops, the themes “my health,” “daily activity,” and “sleep” were established, which corresponded to the most desired functions among the participants to implement in the CA. The analysis focused on identifying and reporting themes and patterns within these 3 functions. This was not based on coding or specific prevalence of the themes identified but through addressing overarching themes of relevance for the 3 most desired functions addressed by the participants [18]. Finally, the thematic analytical approach was inspired by Borgnakke [19], to strengthen the innovative and thematical analytical approach, inspired by practice-oriented research methods.

All researchers took an active part in processing the data, enhancing the process of addressing possible blind spots, and understanding several ways to see the data [20]. In this process, the researchers also acknowledge their active role in the identification, selection, and presentation of themes and patterns from the data in this study [18].

The thematic analytic approach corresponds to the interest in strengthening the relationship between technology development and the participant’s needs and preferences, as well as in cocreating digital health technology that can support older adults’ healthy aging and everyday life. By starting technology development from the participant’s preferences and needs, focusing on meaning-making rather than on adopting a consensus-driven approach, the thematic analysis can help sharpen the sense of themes, problems, and dilemmas seen from the older adult’s perspective, reasons, and relevance criteria. With this analysis, the aim was to capture the relevant themes and layers based on the participants’ thematizations and problematizations and thus increase insight into everyday use situations and challenges.

The transcribed and translated data have not been presented to the participants, and member checking of the analysis has not been conducted [21]. However, due to the iterative approach in the cocreation workshop process, the findings were addressed and validated through the 3 ongoing workshop phases.

## Results

### Overview

In this section, results from the thematic analysis of data from 11 cocreation workshops are presented. The purpose of the analysis was to acquire knowledge and insight into older adults’ own experiences and formulations on important topics and dilemmas connected to their everyday lives in the development of the CA. The results are summarized according to the 3 embedded functions in the CA: “my health,” which addresses the user’s health condition; “daily activity,” which informs about both physical and social activities; and “sleep,” which addresses circadian rhythm.

### My Health

My health was one of the top-rated functions from the beginning of the cocreational process. The participants argued that their health is the most important thing they have. One of the first things addressed by some of the participants was that they want to learn more about their health condition, that is, they want to increase their health literacy. Examples included general information about COPD, for example, “when were you diagnosed with COPD?” “What are the different levels of COPD?” and other commonly asked questions that could be provided to them through the CA. Doing inhalations correctly was also highlighted as an important aspect of their daily life with COPD. Therefore, participants suggested that the CA could contain guides, written or video, on how to do inhale measurements.

An important thing [in relation to my health] is inhalation-technique...it is critical that a patient inhales correctly and is measured on whether you get it down where it needs to be...you can look up some videos on YouTube, they can show it...if there was a video link to a nurse and the citizen had a whistle at home. Then I am sure that it could happen on a video that the person sat and breathed, and the nurse instructed.

M1

An important aspect of the CA development and the function “my health” was that the participants raised concerns about the technology replacing their contact with health care professionals. A lot of the participants were positive toward new technologies in general, and over half of them already used different types of technologies in their everyday life including smartwatches, smartphones, and in-home measuring tools for their condition, for example, technologies provided to them by PreCare. But if a technology was implemented to replace their contact with health care professionals, they were not interested in it.

Well, I absolutely don't agree that technology is a good thing [if the idea is to replace human visits], because my daughter works in healthcare, and she said they're developing things now so they can call you and have this setup where they can check if you have a fever and see everything. What about the poor old folks at home who are isolated? Maybe the home care personnel are the only person they see?

M2

I was also offered when I came into PreCare that I could have a conversation on that tablet, right, then I said I wouldn't agree to that. If I'm going to have a conversation, it has to be face-to-face...but I may be old-fashioned.

F2

The participants mostly trust information from health care professionals. However, they were open to reading health-related information through the CA but required that this information refers to a trusted site, such as *medicin.dk* or *sundhed.dk* in Denmark and *Folkehelseinstituttet* or *Helsenorge* in Norway.

The participants had different needs and interests about the extent and frequency of monitoring their condition. Some participants were interested in measuring a few health data, for example, blood pressure and oxygen saturation, and some were interested in measuring more. Moreover, some participants were interested in measuring their condition daily, and some preferred measuring whenever they felt like it.

I do measurements as much as I can remember to do it...and I feel good about that.

M2

I do measurements every day.

F2

I do my saturation measurements at about 10 or 12...then I can see if the measures are not bad...or think why I am feeling the way I am, oh yes, I can see that on the measurements. That is actually a good indicator for me...

M2

Arguments for wanting to measure their condition daily included getting a feeling of insight into their condition and a general feeling of being aware of their current health state. Arguments against daily measurements included being reminded about their condition and having to identify with it daily. This shows the conflicting feelings and needs when being a patient who receives treatment by the health care system and a person living everyday life with a chronic condition.

I feel a bit ambivalent about it because it annoys me so much that I have to measure my condition every day because then I become aware of it and sometimes, I can't quite get it to hit the numbers I want to. I try again and then it gets even worse and then I get irritated and stop.

F2

The different preferences in terms of usage led to a discussion on how to personalize the CA. The participants suggested a “my profile” function that can help personalize the CA and add or remove measurement units as wanted. This function could also hold data such as height, weight, and information on current medicine intake, etc, that can be entered if relevant.

The participants also suggested the option of sharing their data stored in the CA with health care professionals and informal caregivers. This statement was however not shared by all participants; some found it very relevant, to be able to share data with family or their general practitioner, and some found more value in the data being private, as they did not want to worry or burden their loved ones.

In sum, the “my health” function is highly relevant to several workshop participants, as they state that “our health is the most important thing we have.” The relevance of the function is highlighted in the participant’s wish for more information and knowledge about their condition, guides to do proper in-home measurements daily and comprehensive measurements of different parameters, and the possibility of sharing this data with health care professionals and informal caregivers. However, not all participants shared this view fully, and nuances were identified. Some participants did not wish to measure daily, some wished to measure selected parameters, and others did not want to share their data with others. Therefore, the need for personalization of the app in a “my profile” function was identified.

### Daily Activity

The daily activity function was created as a merge of the 2 top-rated functions—physical activity and social activity—based on ongoing input from the workshop participants. In a brainstorming session, participants suggested that the “daily activity” function should be able to suggest social activities in the local area; motivate them to be active, for example, through text or voice reminders; be able to monitor their activity, for example, through step count; and have an incorporated walking community feature, as some participants found it motivating to walk with others.

I have a “walking-friend”...and we walk together once a week, that could be a thing? Maybe that could be a part of this [the technology].

M1

It [the technology] could for example suggest you contact your walking friend.

M2

Some participants also suggested incorporating knowledge or management of lung capacity in this function, both to learn about how much physical activity they can do in a day and for how long based on their individual condition to feel safe when going outside, as well as inspire easy physical exercises and activities at home.

For the social activities, participants proposed receiving suggestions of social activities in the local area; receiving reminders about activities, for example, pop-up reminders or through a calendar function; or inspiring them to attend social communities for example, “COPD friends,” knitting clubs, and lung choirs.

It’s about knowing what is happening in the local area. I started in a knitting club, and I can’t knit, and I am probably 20 years younger than the other people there, but I have fun, then I just draw or do pearls. It’s the social aspect, and just getting out of the house.

F1

Participants suggested merging physical and social activities, among others, because the participants had different needs and preferences related to physical activity. Some participants had a high activity level and associated physical activity with exercising in a gym. They wanted reminders on planned workout sessions, motivational messages, and inspiration for different exercises; wanted to use sensors, wearables, or smartphones to track their progress; and suggested features such as “my goals” and “my progress.”

It’s a bit funny when you now have the option [to count steps] I catch myself getting annoyed when I've forgotten my phone when I've gone places, then you don't know how much you've walked.

F1

You somehow get happy when it says “you did it”...that means something.

F2

The other group of participants had a lower activity level and associated physical activity with taking out the trash or cleaning the house. This group was also open for activity tracking and goal setting, but also highlighted adding breathing exercises for people who use oxygen supply.

The function could be called daily activity, because doing the dishes or something else, everything counts, anything that gets you out of the couch....it is an exercise just going to the bathroom, at least for me it is, everything counts, so you don’t feel like you are not doing anything at all.

F2

The participants also emphasized that physical and social activities for them are often connected, since just getting out of the house or going for a walk with someone covered both functions. Also, some participants struggled to see how the CA could add value when it measured the same things that they already did on their smartphones, for example, step count. Therefore, the social activity function was appealing to a lot of participants, as they did not have a lot of experience in using technology to find social activities. The daily activity function should ideally according to the participants be able to suggest social activities, both online and physical, for example, cinema, theatre, concerts, lung choir, etc. This would motivate and inspire them to do new things and preserve or boost their social life. A calendar function was suggested to help them keep track of upcoming activities, including social activities, daily household activities, physical activities, doctors’ appointments, medicine, etc.

In sum, both social and physical activities are important to several of the workshop participants. The participants had different needs in relation to physical activities, as some had a high activity level and some had a lower activity level according to their condition and personal wishes. Participants were open to receiving motivational SMS text messages, but this should be optional; thus, the need for personalization of the CA is still relevant for this function. The participants highlight that physical and social activities, for them, are widely connected and therefore wanted input on social activities and gatherings in the neighborhood. The social activity element adds value to the function, as some participants already measured their steps daily through other technologies but were not familiar with using technology to initiate social activities.

### Sleep

The sleep function was well discussed from the beginning of the workshop phases. Some participants did not think that the function was relevant, and others claimed sleep quality to be very important. For the thematic analytical framework, it is important not to stop at this finding. The technology development depends both on the findings of the different opinions and on the specific reasons given by the participants. Those who found it important that “sleep” was included in the CA development justified it with a meaningful aspect, which was “if you don’t sleep well, your whole next day is ruined.”

It [sleep] is important for how you feel during the day, that you sleep well. Personally, I often sleep like hell, and then the day after is ruined. Then you can’t get anything done the next day...

M1

But what this simple justification refers to is, in the perspective of everyday life, a large and complex problem. If the series of weekdays is ruined by poor sleep, then the seriousness and magnitude of the reason are understood. Participants asked for ideas and help to fall asleep, as this was often a struggle. When it comes to measuring sleep, participants had mixed opinions; some stressed that they did not need technology to tell them that they had a poor night’s sleep, and others already used a wearable such as Fitbit (Google) to track their circadian rhythm. Something that was further stressed by this discussion was that the measurements should not be forced; they should be based on need and interest, and not mandatory day-to-day measurements.

I know how much I sleep and whether I’ve slept, I don’t need that.

F1

I think it is probably very different...because there are many who have a little bad sleep, so if they could find out why you have it then you can do something about it, because it means a lot for the whole day, if you have had a bad night, you have a bad day too.

F2

Meanwhile, some participants said that they have gotten the right medication and therefore sleep was not something they worry about. Another participant highlighted that if he wakes up during the night, this is not an issue for him as he is retired and can just sleep in if he wants to. Another participant says that this feature could be useful—only if the CA could tell him when he needs to consult a professional for help solving problems related to sleep, instead of him trying to solve it himself.

Together, these descriptions are illustrative of the heterogeneity of needs in relation to sleep. This may indicate that the sleep feature may need a longer co-design process to be able to catch all nuances that evolve as the participants learn about what the technology may do for them. Specifically, the following features were suggested for the sleep function: advice on how to fall asleep, advice on when to consult a medical professional, reminders to wake up and get oxygen, information on how to get better sleep quality, information on the impact of different medicines on sleep, and ideas for calming breathing exercises.

By starting with the older adults’ reasons and preferences, the thematic analysis sharpens the sense of proportion. Next, the practical suggestions linked to the technical aids or informative COPD-related details allow themselves to be placed with greater precision in relation to the older adults’ perception of technological measures as desirable or not, or useful or useless in everyday use.

Importantly, measurements of sleep were raised several times in conjunction with discussions about this feature. These discussions highlighted that the participants had mixed feelings toward measurements. All participants imagined that such measurements could be useful, but that it must also be optional.

## Discussion

### Principal Findings

In this section, the principal findings based on the 3 themes—my health, daily activity, and sleep—are summarized and discussed in relation to the overarching identified themes, measurements, and health literacy.

The workshop participants expressed strong interest in the “my health” function; some asked for comprehensive information and knowledge about their health guides to proper in-home measurements, highlighting the interest in increasing health literacy, which was identified as an overarching theme, and the ability to share data with health care professionals and informal caregivers. Measurements were also identified as an overarching theme, and it received mixed input; the participants had different preferences regarding the frequency and capacity of measurements. Therefore, personalization of the CA is necessary, which can be achieved through a “my profile” function enabling individualized setup.

Participants emphasized the importance of social and physical activities within the “daily activity” function. Different needs were identified, as some had a high activity level and others had a low activity level due to their condition. Personalized motivational messages were welcomed optionally. The participants highlighted that physical and social activities for them are widely connected; therefore, social activities and gatherings in the local area to get them out of the house were requested. Social activities added increased value to the function, as some participants already tracked their steps daily through other technologies.

Participants had a mixed interest in the “sleep” function. They argued that if you do not sleep well, your whole day is ruined. Some participants also wanted information on how to fall asleep easier, sleep patterns, and tips for better sleep. Measuring sleep was debated, with some finding it useful and others invasive if not optional.

### Measurements

SMILE focuses on older adults’ needs and preferences in undertaking a cocreational approach to technology development. The older adults in the project are participants in developing the CA, patients with chronic conditions, citizens in a community, and users of the CA. As identified in the workshops, not all participants want to be reminded that they are patients every day, for example, through daily measurements, and a lot of emphasis was placed on the importance of social activities being a part of the CA. However, with the implementation of new health technologies, the borders between private homes and the health care system are torn down, including technologies entering citizens’ homes, enabling daily measurements.

Measurements are a cross-cutting theme and a common focal point for the SMILE project’s technology development and for the workshop participants’ decision on technological aids. Daily measurements seem to be an important feature that can optimize and aid participants through, for example, novel health technologies, such as smartwatches. However, daily measurements did receive mixed interest. The workshop participants highlighted that being measured every day, as a person living with COPD, is for some valuable and others harmful. Regardless of this, measurements must be seen in conjunction with other themes, such as physical activity. Some participants already measured their physical activity through Fitbit or on their smartphone (eg, step count).

Moreover, the participants who already monitored their steps daily doubted how the CA could bring them further value. The participants also emphasized that measurement must be easy to do and ideally automatically, as with smartwatches. Participants were open in using one solution, such as the CA, to do daily measurements; if it could collect all the measures, they found valuable as they did not have to measure everything twice.

Based on this, it can be emphasized that measurement must be needs based. Not all participants are interested in measuring several parameters every day. For some participants, the motivation to measure is that they feel ill. During those periods, they feel the need to monitor themselves. Others are motivated to do measurements to follow their condition daily. Regardless, it is important to emphasize that the main attitude of the workshop participants toward the CA was formulated almost as a statement: whatever may be offered to them by technological aids, they do not want it to become a substitute for their face-to-face contact with health care professionals.

### Health Literacy

The long-term aim of the development and implementation of the CA is that it can be used by citizens in their own homes, regardless of health conditions and independently of social and professional networks. Studies have addressed how digital solutions and a focus on more hybrid care models have the possibility of relieving the global burden on health care systems and health care staff [22]. However, these digital solutions need to be perceived as valuable by several actors in the specific health care setting and need to be used “correctly” to prevent adding an extra burden on the staff and to enable widespread adoption among patients [23].

As illustrated in the analysis, the participants are interested in learning more about their health, tracking their physical activities, and getting advice and techniques to improve their sleep. In addition, they are interested in reflecting on their life situation as older adults living with COPD, from an everyday perspective.

At the same time, the thematization possibilities can be seen in relation to the development of professional education, which includes both health skills and digital literacy and deals with cocreational technology development. Theoretical approaches and concepts, such as Negt’s [24] conceptualizations of identity and technological competence, are relevant and can be put into the perspective of the themes and issues raised by the workshop participants. Negt’s statements that “identity competence relates to a lifelong learning process” and that “identity is not determined once and for all” resonate in relation to the results from the first phase of the workshops. In relation to the focus of SMILE and the workshop participants, this means that the orientation and perspective of the participants are challenged, partly by what Negt [24] describes as a reality test between “the well-ordered society” and “the marginalized environments, groups” and partly by the development of health literacy as a, didactically speaking, lifelong interaction among case, knowledge, and experience. Concerning technological competence, the challenges refer both to the development of an ability to distinguish between technologies and to the understanding of technology as a social project. In SMILE auspices, it also refers to the dilemma between the technology development system and patient orientation, with the latter guided by human needs, closer to the participant’s everyday life and experiences.

To clarify the technological user value, there is an implicit demand that older adults with COPD have some degree of both health literacy and digital literacy. Correspondingly, it requires a similar and explicit demand that health professionals have digital literacy, not only in terms of individual competence but in terms of collaborative and patient-oriented competence across professions. Ethnographic studies close to health care practice show examples of patient-oriented interprofessional cooperation as well as how interprofessional competence development can support the bridging between the hospital context and the home context [20,21].

Against this background, the results show that participants in the SMILE workshops prefer not to replace their interaction with health care professionals with technology but are open to using it as an additional tool in care. Thus, if the CA is able to inform the users about health-related topics, it needs to be from trusted sources. The participants emphasize that health care professionals must be active contributors to address health challenges alongside technology.

### Conclusions

Future development of digital health services should encompass the complexities of everyday life among older adults to enhance usability, adoption, and successful use. Participants in the SMILE workshops are interested in reflecting on their life situation as older adults with COPD from an everyday perspective. Participants are open to using new technology, the CA, to learn about their health, monitor physical activity, and discover social activities in their local area. The CA should not only focus on the “patient” and the condition but also acknowledge the complexities of everyday life as an older adult living with COPD. This means grasping the different needs and preferences of individuals, regardless of their condition, in designing technology is important. Daily measurements should be optional, and digital solutions should be used as a supplement, not a replacement for in-person interaction with health care professionals.

## Appendix

Multimedia Appendix 1 Consent form.

## Abbreviations

CA: conversational agent
COPD: chronic obstructive pulmonary disease
READHY: Readiness and Enablement Index for Health Technology
SLE: smart living environment
SMILE: Smart Inclusive Living Environment
WHO: World Health Organization

## Data Availability

The datasets used and/or analyzed during this study are available from the corresponding author on reasonable request.
