# Supplementary material for: Co-Designing a Conversational Agent With Older Adults With Chronic Obstructive Pulmonary Disease Who Age in Place: Qualitative Study
Source: JMIR Hum Factors. 2024 Oct 8;11:e63222. doi: 10.2196/63222 (PMC11496918; doi:10.2196/63222)
Supplement: Multimedia Appendix 1 [file humanfactors_v11i1e63222_app1.docx]

### **Multimedia appendix 1**

### Consent to the processing of your data in a research project

Thank you for participating in the SMILE project and participating in the University of Copenhagen's study focusing on citizens' needs in the development of technology to be used in their own homes in connection with managing their health.

We need your consent for our research group at the University of Copenhagen to process the health and personal data we collect concerning this study. We ask for your consent according to the rules of the General Data Protection Regulation.

We will collect data in the form of workshops, interviews, photos, descriptions of conversations, and ideas explained by you.

Your data will be stored on a secure and approved server at the University of Copenhagen and will be deleted after completion of the project and no later than the end of 2029. Data may be shared with Region Zealand and researchers at the Local Clinic, as well as in anonymized form with the other partners of the SMILE project.

You can at any time be informed what data is registered about you and who processes it and has access to it.

**Title of the project:**  SMILE 'Smart Inclusive Living Environments'

- I confirm that I have read and understood the above information.
- I hereby consent to the University of Copenhagen collecting, registering, and processing my health and personal data in the above-mentioned research project.
- I am informed that I can withdraw my consent at any time, that it will not reduce my opportunities to receive usual health and treatment offers, and that my data will be deleted to the extent that they are not anonymous or included in aggregate data if I withdraw consent.

Name: __________________________________________

Date and signature: _______________________________

I consent to the health and personal data collected here being shared with other research institutions participating in the SMILE consortium in Denmark or another country within the EU/EEA,

You can withdraw your consent by contacting research assistant Emilie Wegener, by mail, and telephone.
